# A fully automated binning method for improved SHARP reconstruction of free-breathing cardiac images

**DOI:** 10.1186/1532-429X-18-S1-P267

**Published:** 2016-01-27

**Authors:** Aurelien Bustin, Freddy Odille, Guido P Kudielka, Martin A Janich, Anja C Brau, Anne Menini

**Affiliations:** 1 GE Global Research, Freising, Germany; 2 Department of Computer Science, Technische Universität München, München, Germany; 3 Universite de Lorraine, Imagerie Adaptative Diagnostique et Interventionnelle, Nancy, France; 4 Inserm, U947 Nancy, France; 5 Cardiac Center of Excellence, GE Healthcare, Garching, Germany

## Background

Despite recent progress in fast cardiac imaging, respiratory motion remains a challenging problem, usually leading to poor image quality when scanning poor breath-holder patients or acquiring high spatial resolution images. Today respiratory motion is compensated using navigators or external physiological sensors and can result in decreased scan efficiency and increased setup complexity. We recently proposed a motion compensated reconstruction, Single-sHot Accelerated Reconstruction with Preserved-features, or SHARP, that enables high-resolution motion-corrected reconstruction of multiple single-shot images acquired in free-breathing, with respiratory motion derived directly from the single-shot images. In the present work, a fast and automatic self-navigated binning method is described, which aims to accelerate the SHARP reconstruction process while improving image quality. The rationale for accelerating SHARP is that raw data acquired in similar motion states can be clustered into a reduced number of motion states, thereby, improving the quality of images from which to extract motion.

## Methods

**Figure 1:**
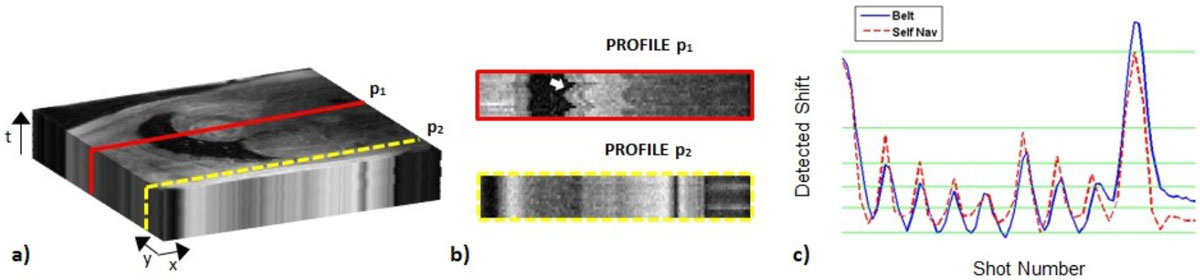
**Schematic illustration of the method**. Low-res images are stacked along the time dimension (a). One cross-section through the 3D spatial-temporal volume shows a 2D image in the x-t plane (b). If the pixels are static over time (profile p_2_), the x-t image would exhibit horizontal and vertical structures, while a profile going through the myocardium (profile p_1_) with moving objects along the time dimension, would show more inhomogeneous structures, thus generating a pattern with breathing motion information (c). The extracted respiratory signal (red) shows good agreement with the respiratory belt (blue), with a coefficient of determination R^2^ = 0.76.

Accelerated single-shot cardiac-gated fast gradient echo imaging was performed on a 3T MR750w system (GE Healthcare, WI, USA) on four healthy volunteers during free breathing. Interleaved golden-ratio k-space acquisition pattern was used, as previously described. For comparison, a breath-hold scan was acquired with the same sequence. Low-resolution images were obtained from the k-space center of each shot and were stacked together along the time dimension. The moving parts of the image were automatically extracted by band pass filtering along the time-dimension with the frequency interval [0.05, 0.5] Hz corresponding to the frequency range of respiration. The motion mask was obtained by summing over time and thresholding the back-transformed volume. A singular value decomposition (SVD) was then applied on the 2D mask (Figure 1).

## Results

**Figure 2:**
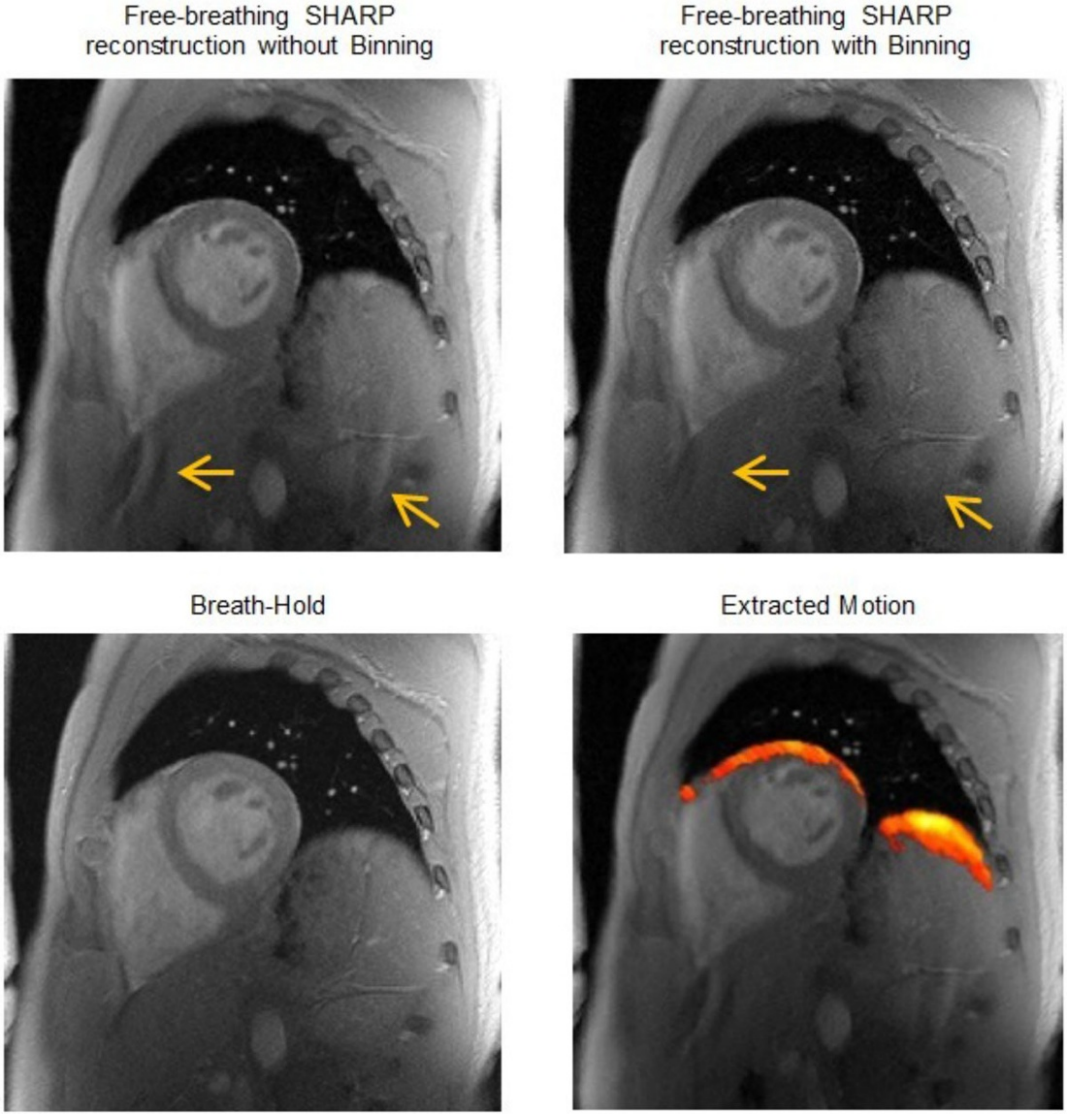
**Cardiac imaging reconstructions with automatic binning and SHARP reconstruction after 15 single-shot SPGR acquisitions in free breathing (breath-hold and free-breathing scan durations were approximately 6 s)**. A significant visual improvement as well as a reduction of artifacts is seen when correcting for the motion using respiratory binning, compared to motion compensation only (arrows). The time needed to run the motion-compensated reconstruction for 15 shots of matrix size equal to 192 x 256 with an 18-ch cardiac coil was about 4 min 52 seconds and is reduced to 1 min 35 seconds when the data are clustered into 5 bins.

The best fit line representing the temporal stack was the first column of the left-singular vectors which corresponds to the highest singular value. An estimate of the true respiratory signal is obtained from this vector. The algorithm is computationally inexpensive since it only requires filtering and SVD of a stack of 2D small patches. Now given the breathing signal, acquired data are summed according to similar position in the respiratory cycle, therefore reducing the amount of data shots (Figure 1). The recent SHARP reconstruction is applied to compute a motion-free image (Figure 2).

## Conclusions

The proposed method allows the extraction of a respiratory signal directly from the acquired images, providing a way to reconstruct faster, higher-quality single-shot images in free-breathing without the need for navigators or external sensors. The next planned step is to apply the method to clinical applications, such as pediatric or severely ill patients, in which breath-hold requirement is challenging and yet high-quality, high-resolution imaging is still required.

